# Fallen Angels or Risen Apes? A Tale of the Intricate Complexities of Imbalanced Immune Responses in the Pathogenesis and Progression of Immune-Mediated and Viral Cancers

**DOI:** 10.3389/fimmu.2014.00090

**Published:** 2014-03-06

**Authors:** Beatrice Omusiro Ondondo

**Affiliations:** ^1^The Jenner Institute, Nuffield Department of Medicine, University of Oxford, Oxford, UK

**Keywords:** regulatory T cells, immune dysfunction, immune-regulation, inflammation, cancer, HIV-1

## Abstract

Excessive immune responses directed against foreign pathogens, self-antigens, or commensal microflora can cause cancer establishment and progression if the execution of tight immuno-regulatory mechanisms fails. On the other hand, induction of potent tumor antigen-specific immune responses together with stimulation of the innate immune system is a pre-requisite for effective anti-tumor immunity, and if suppressed by the strong immuno-regulatory mechanisms can lead to cancer progression. Therefore, it is crucial that the inevitable co-existence of these fundamental, yet conflicting roles of immune-regulatory cells is carefully streamlined as imbalances can be detrimental to the host. Infection with chronic persistent viruses is characterized by severe immune dysfunction resulting in T cell exhaustion and sometimes deletion of antigen-specific T cells. More often, this is due to increased immuno-regulatory processes, which are triggered to down-regulate immune responses and limit immunopathology. However, such heightened levels of immune disruption cause a concomitant loss of tumor immune-surveillance and create a permissive microenvironment for cancer establishment and progression, as demonstrated by increased incidences of cancer in immunosuppressed hosts. Paradoxically, while some cancers arise as a consequence of increased immuno-regulatory mechanisms that inhibit protective immune responses and impinge on tumor surveillance, other cancers arise due to impaired immuno-regulatory mechanisms and failure to limit pathogenic inflammatory responses. This intricate complexity, where immuno-regulatory cells can be beneficial in certain immune settings but detrimental in other settings underscores the need for carefully formulated interventions to equilibrate the balance between immuno-stimulatory and immuno-regulatory processes.

## Introduction

The observation that a sustained and potent immune response to a foreign pathogen, self-antigen, or normal microflora can be the root cause of uncontrolled cancer outgrowth and progression underscores the need for tight immuno-regulatory interventions that could be harnessed for the development of cancer vaccines and cell-based immunotherapies. On the other hand, inflammatory responses characterized by infiltration of tumor-associated antigen (TAA)-specific T cells and other components of the innate immune system are a pre-requisite for effective anti-tumor immunity. Therefore, it is crucial that the inevitable co-existence of these opposing forces is carefully streamlined as imbalances can be detrimental to the host.

Oncogenic viruses such as Epstein–Barr virus (EBV), human papilloma virus (HPV), and Kaposi sarcoma herpes virus (KSHV) express viral oncogenes, which can directly induce tumorigenic cell transformations and initiate the carcinogenesis process. In the case of non-oncogenic viruses such as hepatitis B (HBV) and hepatitis C (HCV), chronic infection and inflammation can lead to carcinogenic mutations in host cells (1), which are manifested by the increased incidences of liver cancer in chronic HBV and HCV patients. In both of these scenarios, the arising transformed tumor cells are genetically altered in a manner that distinguishes them from ordinary healthy self-cells thus conferring the ability to trigger effector immune responses, which in some cases are capable of controlling tumor growth (2, 3). In other instances, however, such modifications may lead to altered antigenicity and escape from immune-surveillance whereby the newly transformed cells are no longer recognized by their original cognate antigen-specific immune cells, thus leading to uncontrolled cancer progression. On a different platform, continuous antigenic stimulation that occurs during chronic virus infections causes severe immune dysfunction characterized by T cell exhaustion, anergy and in some cases deletion of antigen-specific B and T cells (4–6), and a concomitant induction of immuno-regulatory processes, which all result in the loss of tumor immune-surveillance and lead to cancer establishment. This is indeed supported by epidemiological data showing increased incidences of malignancies such as Kaposi sarcoma (KS) and cervical cancer, as well as EBV-associated malignancies such as non-Hodgkin lymphoma (NHL) and Burkitt lymphoma in immunosuppressed HIV/AIDS (7) and transplant patients.

Cancer can also arise due to dysfunctional immuno-regulatory mechanisms that result in uncontrolled excessive inflammatory immune responses. For example, pathogenic immune responses directed at commensal intestinal microflora during inflammatory bowel disease (IBD) are known to increase the risk of colon cancer (8, 9). Indeed prolonged periods of ulcerative colitis (UC) and Crohn’s disease (CD) are associated with impaired immuno-regulatory mechanisms, which are in turn linked to colitis-associated colon carcinogenesis (10–12). Under normal circumstances both intrinsic and extrinsic regulatory pathways come into force to limit excessive immune activation and inflammation thus preventing tissue pathology and subsequent risk of cancer. However, as in many cases, failures of these control measures, including reduced frequencies or altered phenotype and function of regulatory T cells (Treg) means that this inflammation progresses in leaps and bounds. These paradoxical scenarios highlight a disruption in the natural homeostatic immuno-regulatory mechanisms that can be switched on to prevent excessive immune activation or turned off to allow execution of effector immune responses and tumor immune-surveillance. However, the exact timing of when a “good” immune response aimed at pathogen or tumor clearance can become a very “bad” response that creates an environment conducive for cancer growth and dissemination remains elusive. Understanding the intricate complexities and the timings of these events will be crucial in designing interventions for immune-mediated and viral cancers.

## Extrinsic and Intrinsic Immuno-Regulatory Pathways

**Figure 1:**
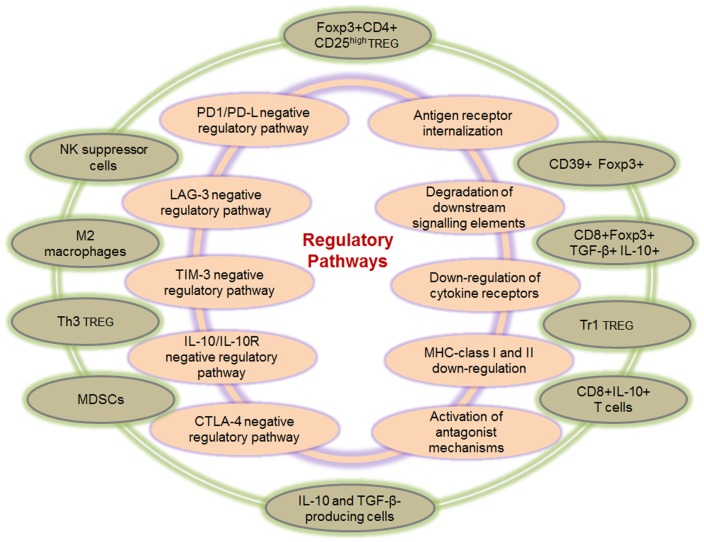
**Intrinsic and extrinsic immune-regulatory pathways**. Several pathways of immune-regulation exist, and these comprise intrinsic and extrinsic mechanisms. Intrinsic pathways (inner circle) derive from within the effector cell and usually involve up-regulation of inhibitory receptors, down-regulation of cytokine and T cell activation receptors, down-regulation of MHC molecules, as well as the degradation of downstream signaling elements. Although the intrinsic pathways derive mainly from within the effector or antigen presenting cells, interactions with external elements do play a significant role, for instance the down-regulation of MHC class I, which is directly mediated by the HIV-1 Nef protein (13, 14). Extrinsic pathways (outer circle) involve several other cell types that exert immune suppression via cell-to-cell contact or through the release of suppressive cytokines and other biochemical compounds with suppressive activity. These include the various types of regulatory T cells in addition to the Foxp3+ Treg, CD8+ regulatory T cells, MDSCs as well as M2 macrophages and suppressive NK cells.

A complex network of finely tuned immune-regulation pathways exists to actively inhibit excessive immune responses during chronic viral infections and inflammation. This is essential for preventing the hyper-proliferation of antigen-specific T cells that could cause immunopathology due to increased release of inflammatory cytokines and targeted killing of infected or antigen-expressing tumor cells by CD8+ T cells. Immuno-regulatory pathways can broadly be divided into extrinsic or intrinsic pathways as depicted in Figure 1. Intrinsic mechanisms derive from within the effector cell and usually involve down-regulation of activating receptors and up-regulation of inhibitory receptors as well as activation of antagonist mechanisms, as discussed in the next section. Extrinsic pathways on the other hand usually involve other cells, which exert regulatory functions by cell-to-cell contact or via release of suppressive cytokines and biochemical compounds that inhibit cellular functions.

Of the extrinsic immuno-regulatory pathways, CD4+CD25^high^ Foxp3+ Treg are the most extensively studied and their suppressive mechanisms have been elucidated in greater detail. Existence of other types of immune cells with regulatory functions has been documented, for example CD4+Foxp3− Treg with suppressor functions such as the IL-10 producing Tr1 cells (15) and TGF-β producing Th3 cells (16) have been found in inflammatory environments. Tr1 cells secrete high levels of IL-10 and moderate amounts of TGF-β, and mainly suppress vial IL-10 release, as IL-10 neutralization abrogates their suppressive function (17, 18). On the converse, Th3 cells secrete high levels of TGF-β and low levels of IL-10 and can suppress both Th1 and Th2 responses (16, 17). Other cells with regulatory properties include myeloid-derived suppressor cells (MDSC), which can be induced by cytokines such as IL-6 and growth factors including G-CSF and GM-CSF (19), CD8+Foxp3+ Treg producing both IL-10 and TGF-β, or IL-10-producing CD8+ T cells (20–22), as well as NK cells that possess suppressor functions (23, 24). Activated MDSC can suppress via several mechanisms including IL-10 production as well as via compounds such as arginase 1, reactive oxygen species (ROS), and nitric oxide (NO) among others (19). Moreover, MDSC can indirectly contribute to immuno-regulatory functions by inducing Treg differentiation and expansion.

## Immune Dysregulation during Persistent Virus Infections and Chronic Inflammation

**Figure 2:**
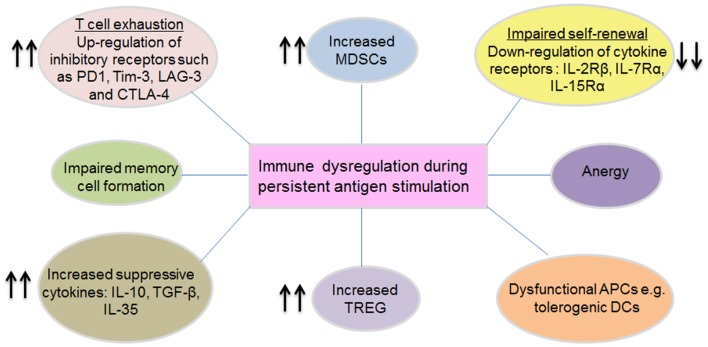
**Mechanisms of immune dysregulation during persistent antigen stimulation**. Immune dysregulation manifests in several distinct forms, which can occur in isolation or in combination. Persistent antigenic stimulation, especially in settings with high antigen loads can lead to T cell exhaustion (characterized by the up-regulation of several inhibitory receptors and down-regulation of specific T cell activation receptors), anergy (generalized unresponsiveness), impaired memory formation, impaired proliferation, and self-renewal capacity. Besides these, chronic viruses trigger various biochemical pathways that lead to increased frequencies of MDSCs and Treg, which actively suppress effector immune responses via a variety of mechanisms. Immune dysregulation occurring at the T cell priming stage is linked to dysfunctional APCs (for example inadequately activated or tolerogenic DCs), which are likely to skew the immune response toward tolerance. Conceivably, such regulatory mechanisms serve to prevent tissue damage and aberrant immune activation, but they inevitably contribute to the chronic persistence state as a result of inhibiting effector immune responses.

T cells are the key players in many infectious diseases and in eradication of malignant cells. This is well-demonstrated in acute infections where T cells become activated and acquire effector functions, with subsequent clearance of infection and formation of stable memory populations. Moreover, tumors heavily infiltrated with fully functional effector T cells progress less rapidly and in some cases regression can be achieved. However, in the case of persistent antigen stimulation in a chronic setting, memory T cell formation and effector functions are altered, resulting in exhausted, functionally impaired defective T cells incapable of conferring protection. The characteristic properties of these defective cells include diminished cytokine production, decreased cytotoxicity, and reduced proliferative and self-renewal potential. In some cases, mutational escape and/or physical deletion of antigen-specific T cells occurs resulting in inadequate immune control, hence chronic persistence of the viruses. Furthermore, some chronic pathogens directly infect the immune cells, e.g., HIV-1 (CD4+ T cells) and EBV (B cells) leading to loss of immune functions. This state of immunological dysfunction is consistently found in chronic virus infections including HIV, HBV, and HCV (25–27) and is also prevalent in cancer patients. Immune dysregulation can be manifested in several forms, some of which are summarized in Figure 2 and described below.

### T cell exhaustion

T cell exhaustion refers to a state of progressive loss of immune function, which in some cases, can result in physical deletion of responding cells due to imbalances in the expression of pro-apoptotic and anti-apoptotic factors and the inability to respond to IL-7 and IL-15 (26–28). The dominant mechanism of T cell exhaustion is the up-regulation of several inhibitory receptors, although down-regulation of cytokine receptors such as IL-7Rα and IL-15Rα by exhausted memory T cells is frequently observed. Lower levels of IL-7Rα and IL-15Rα can lead to defective cytokine signaling and consequently impaired homeostatic self-renewal and suboptimal numbers of functional memory T cells (27, 28). Up-regulation of inhibitory receptors such as programed-death 1 (PD-1), T cell immunoglobulin mucin 3 (TIM-3), lymphocyte activation gene 3 (LAG-3), and cytotoxic T-lymphocyte-associated protein-4 (CTLA-4) is a characteristic feature of exhausted T cells. PD-1, an inhibitory receptor of the CD28 superfamily is highly expressed on exhausted CD8+ T cells during progressive chronic viral infections and uncontrolled cancer, making it a major factor in T cell exhaustion. Under normal circumstances, PD-1 is induced following T cell activation to inhibit the TCR signaling cascade and prevent excessive T cell activation, but is then down-regulated following pathogen clearance. In peripheral tolerance, PD-1 is important in inhibiting potentially pathogenic self-reactive T cells as well as promoting Treg development (29, 30) and mice lacking PD-1 succumb to autoimmune diseases (31, 32). However, in chronic infection, the PD-1 pathway mediates pathogen-specific CD8+ T cell dysfunction as demonstrated in HIV (33–35), HCV (36, 37), and HBV (38, 39) infections. For example, the frequency of PD-1+CD8+ T cells is highly elevated in HIV-1 patients where it correlates significantly with viral load and declining CD4+ T cell numbers (33, 40). PD-1 is also up-regulated on HIV-specific CD4+ T cells (40, 41) and inhibits CD4+ T cell responses including proliferation. Interestingly, PD-1 levels are significantly reduced in HIV-1 progressors who initiate highly active antiretroviral therapy (HAART) or in long-term non-progressors (LTNPs), suggesting that antigen persistence drives T cells to exhaustion (33, 34). In chronic HCV infection, increased PD-1 expression on HCV-specific CD8+ T cells is associated with impaired proliferation and cytokine production (37). A part from inhibition of T cell function, PD-1 expression can also lead to spontaneous or FAS-mediated apoptosis of virus-specific T cells (42).

Besides PD-1, other inhibitory receptors such as TIM-3, 2B4 (natural killer cell receptor), and LAG-3 are also up-regulated on virus-specific T cells, and the expression of multiple inhibitory receptors correlates with a severely dysfunctional state (43–45). For example, co-expression of PD-1 and TIM-3 is associated with severely exhausted HIV-specific CD8+ T cells (45) and majority of these also co-express PD-1 and 2B4 (46). CTLA-4 is another inhibitory receptor expressed by activated CD4+ and CD8+ T cells. It has a higher affinity for the B7 ligands (CD80 and CD86) allowing it to out-compete CD28, hence it is a powerful negative regulator of CD28-dependent T cell responses. It is significantly up-regulated on CD4+ T cells during HIV-1 and HCV infections where it correlates positively with disease progression and negatively with antigen-specific IL-2 production (41, 47). CTLA-4 is abundantly expressed on Treg as it is required for optimum suppressive function.

### Impaired APC function

The fact that fully functional pathogen-specific T cells are rarely found in chronic infections suggests impaired antigen presentation, which could be attributed to either inadequate priming by non-professional APCs or possibly altered function of professional APCs during the chronic stages of disease. Indeed, functional impairment of DCs has been associated with T cell exhaustion and progression of disease during HIV, HBV, HCV, and LCMV infection (48–51). Decreased expression of co-stimulatory molecules and lower production of immuno-stimulatory cytokines by APCs can result in functionally tolerant or anergic T cells. Furthermore, chronic infections are associated with loss of DCs, possibly due to direct infection by viruses such as HIV and LCMV. Moreover, DCs can induce T cell exhaustion or tolerance by signaling through inhibitory receptors such as PD-1 and CTLA-4, and also acting via indoleamine 2,3-dioxygenase (IDO)-dependent mechanisms to induce Treg, which further suppress immune responses (52). However, other factors such as virus-induced modulation of the expression of MHC or co-stimulatory molecules have been described and may also significantly affect the generation of fully functional T cells (13, 14).

### Increased frequency of Treg and MDSC

Increased frequencies of Treg and MDSC are a common feature of persistent chronic viral infections, which is well-documented in infections with HBV (53–55), HCV (56–58), and HIV (52, 59–61). These chronic persistent viruses trigger the production of IL-10 and TGF-β, which in turn increase the frequency and suppressive function of Treg, such as observed in HCV-infected hepatocytes (62). Alternatively, these cytokines may promote the induction of adaptive Treg further reinforcing the immune barrier at sites of infection. HIV and HCV infections also induce plasmacytoid dendritic cells (pDCs) known to induce IL-10-producing Treg via IDO-dependent mechanisms (52, 58). Additionally, the chronic micro-environments created by virus persistence contribute to enhanced Treg proliferation and suppressive function by secreting cytokines and other factors on which Treg thrive. The high frequencies of Treg and MDSC serve an important role of preventing excessive antigen stimulation, persistent inflammatory responses, and viral mediated immunopathology in the chronic stages of viral disease (56, 63). However, the elevated frequencies and enhanced suppressive capacity of Treg and MDSC also contribute to suppression of effector T cells in an antigen-specific or bystander mechanism (64) thus promoting prolonged viral persistence (65, 66) characterized by secondary T cell impairment and exhaustion (67). Thus, counterintuitively, increased expansion and survival of regulatory cells serve to establish, propagate, and maintain the chronic infection state.

### Increased suppressive cytokines

Apart from Treg and MDSC, increased IL-10 production is another powerful immuno-regulatory mechanism that negatively impacts on the quantity and quality of antigen-specific immune responses. IL-10 is an immuno-regulatory cytokine produced by many cell types and has multiple functions including inhibition of pro-inflammatory cytokine production, dampening T cell responses, blocking APC functions, and also causing B cell dysregulation. Increased IL-10 production is seen in several chronic viruses including HIV, EBV, HCV, HBV, and LCMV (68–75), and IL-10R blockade can induce rapid virus control indicating that excessive levels of IL-10 have a negative influence on the quality of immune responses and disease course (68, 69). TGF-β is yet another immunosuppressive cytokine whose role in limiting immune responses is documented in a number of disease settings (76). Both IL-10 and TGF-β are known to establish highly suppressive micro-environments that are suitable for cancer progression.

## Disruption of Immune-Regulatory T Cells in Inflammatory Environments

Resolution of inflammation requires swift execution of functional regulatory mechanisms such as the expansion of Treg, a lineage of lymphocytes committed to suppressive functions that maintain self-tolerance and immune homeostasis. Dysregulation of Treg function or induction is linked to a number of chronic inflammatory disorders such as IBD and also fatal autoimmune diseases. Thus, interventions which can restore functional regulation without inducing effector immune responses would be beneficial in such settings. Dysfunctional regulation can manifest as reduced Treg numbers (either due to defective Treg induction or loss of Treg), defective suppressive function (due to loss of Foxp3 expression or reduced production of suppressive cytokines), and impaired migration (due to altered expression of adhesion molecules and chemokine receptors). This section gives a brief description of these mechanisms and the various inflammatory conditions that drive phenotypic and functional modification of Treg.

### Treg instability: Phenotypic alteration and functional impairment

**Figure 3:**
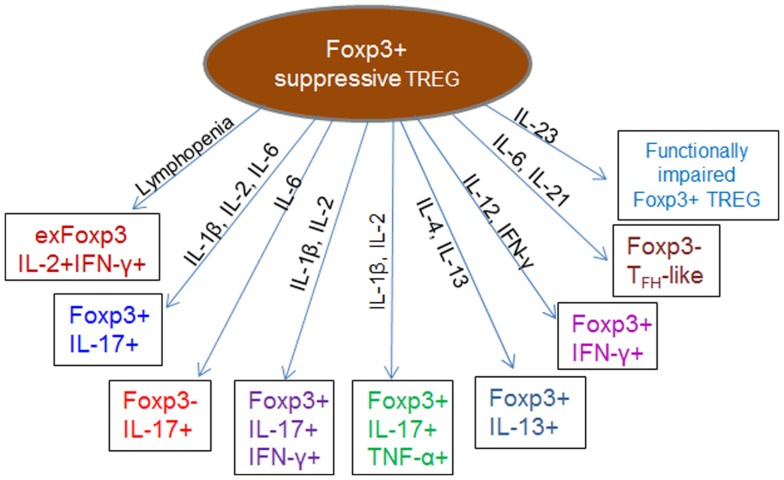
**Impaired or altered function of regulatory cells during inflammation**. The cytokine and chemokine milieu of inflammatory micro-environments can induce phenotypic and functional modification in Treg, leading to generation of pathogenic exFoxp3 T cells, which express lower levels of Foxp3, CTLA-4, and GITR and produce a combination of Th1, Th2, or Th17 cytokines. Conversion of Foxp3+ Treg into pathogenic IFN-γ-producing Th1 cells or IL-17-producing Th17 exFoxp3 Treg cells is documented in various immunological settings (77, 80, 101). Conversion to a Th2 phenotype expressing IL-13 (Foxp3+IL-13+) and IL-5 (78, 83) as well as differentiation into follicular helper T cells (84) have also been reported. In most cases, the suppressive function of these altered phenotypes is significantly reduced due to decreased Foxp3 expression (81, 82). Certain inflammatory conditions can support the generation of hybrid phenotype Treg, which exhibit dual suppressive and pro-inflammatory functions such as the IL-17-producing Foxp3+IL-17+ (85–90), IFN-γ-producing Foxp3+ IFN-γ+ (91, 92), Foxp3+IL-17+ IFN-γ+, or Foxp3+IL-17+ TNF-α+ (102, 103) Treg. Generally, environments enriched with Th1 cytokines such as IFN-γ, IL-2, and IL-12 favor generation of exFoxp3 Treg producing IFN-γ, those enriched with Th2 cytokines such as IL-4 and IL-13 favor generation of Th2 Treg, while IL-6 favors conversion into the IL-17+Foxp3+ and IL-17+Foxp3− phenotypes.

Despite the widely held view of thymic imprinting of Treg cell functions, recent studies indicate developmental plasticity and instability, whereby Treg lose Foxp3 expression and convert to Foxp3− helper T cells (exFoxp3) (77, 78) in certain inflammatory or lymphopenic environments (Figure 3). Although exFoxp3 Treg may largely arise from a few promiscuous uncommitted Treg (79), their comparatively higher potential to expand, coupled with the fact that a majority of them are skewed toward self-reactivity suggests potential pathogenicity as a result of altered regulatory functions such as secretion of pro-inflammatory cytokines directed against self-antigens (77). Adoptive transfer studies showed that a large fraction of Treg transferred to lymphopenic recipients lost Foxp3 expression alongside other Treg cell surface markers, and that this was accompanied by acquisition of effector functions including IFN-γ, IL-2, and IL-17-production and a concomitant loss of suppressive function (80–82). Other reports indicated that Foxp3+ Treg effectively lost Foxp3 expression and converted to T helper-type 2 phenotype cells expressing IL-13 and IL-5 (78, 83) or differentiated into follicular helper T cells (Foxp3–T_FH_-like cells) under the influence of IL-6 and IL-21 (84). Acquisition of T helper features without the simultaneous loss of Foxp3 expression has also been observed. This results in hybrid Treg, which display an activated-memory T cell phenotype and pro-inflammatory properties, such as the IL-17-producing Foxp3+ROR-γt+ IL-17+ (85–90) and IFN-γ-producing Foxp3+T-bet+ IFN-γ+ (91, 92) Treg. Although this hybrid Treg phenotype can exert dual inflammatory and regulatory functions, it has been shown that the phenotypic and transcriptional modifications can reduce their overall suppressive function (81, 93). In other instances however, Treg have been shown to lose their suppressive function without necessarily converting to exFoxp3 or dual function (hybrid) inflammatory Treg (94–100). Such functionally impaired Treg show decreased expression of Foxp3, CTLA-4, and GITR, together with production of very low levels of IL-10 and TGF-β.

Several lines of evidence indicate that functional and phenotypic plasticity of Foxp3+ Treg is largely governed by extrinsic signals provided by the inflammatory milieu of their surrounding environments. Increased levels of pro-inflammatory cytokines such as IL-12 or IFN-γ correlate with the frequency of functionally impaired Th1-like Treg (104). In this setting, the Treg suppressive functions were effectively restored by IL-12 withdrawal or IFN-γ blockade suggesting that a pro-inflammatory cytokine milieu not only promotes the Th1-like phenotype, but also inhibits Treg suppressor functions. Overall, inflammatory environments enriched with cytokines such as IL-1β, IL-4, IL-6, IL-21, and IL-23 drive conversion of Foxp3+ Treg into T helper phenotypes (80, 105, 106). As an example, stimulation of peripheral Treg in the presence of IL-6 was shown to result in loss of Foxp3 expression and production of IL-17 (105, 106). Inflammatory environments with IL-1β, IL-2, IL-6, IL-21, IL-23, and TGF-β have been shown to drive conversion of Foxp3+ Treg into IL-17 producing Treg (87, 107), whereas TGF-β, IL-10, and IL-2 help to maintain continued Foxp3 expression, Treg stability, and suppressive function (80, 81, 92, 108–110). Therefore, stable Foxp3 expression and maintenance of optimal Treg suppressive function require the continuous presence of specific signals within the inflammatory environment, without which conversion of Treg into functionally impaired exFoxp3 T cells or hybrid phenotype Treg occurs (102, 103).

Although the various Treg phenomena described above are well-documented in autoimmune settings, it remains possible that the chronic inflammatory environments created by persistent viral infections can also support phenotypic and functional modifications that would render Treg dysfunctional. In favor of this speculation, a recent study has demonstrated that Treg infected with HIV display increased CpG methylation of the Foxp3 locus and a deregulated functional profile, which was characterized by down-regulation of Foxp3 expression, reduced suppressive capacity, and altered cytokine secretion pattern (111). These Treg showed decreased production of TGF-β and increased IL-4 secretion, a characteristic which is thought to orchestrate severe systemic immune hyper-activation that is observed during progressive HIV disease. In chronic infection with HCV, PD-L1 was found to negatively regulate both the function and proliferation of Treg by controlling STAT-5 phosphorylation (112). Although PD-1 was expressed on both Treg and effector T cells, Treg showed significantly higher up-regulation of PD-1, which was correlated with disease progression. These studies highlight the potential of viruses to subvert the induction and function of Treg, but clearly further research is needed to unravel the mechanisms underlying defective regulation during chronic virus infections.

### Impaired or altered migration of Treg

Another crucial aspect contributing to Treg dysfunction is their ability to migrate to peripheral sites of chronic inflammation such as the skin, urogenital mucosa, gut-associated lymphoid tissues (GALT), transplanted organs, or tumors for appropriate localization, in close proximity with effector immune cells as suppression is mostly contact-dependent. To do this effectively, activated Treg up-regulate distinct site-specific inflammatory chemokine receptors and adhesion ligands, which facilitate their migration into the inflamed tissues, usually in response to a variety of inflammatory chemokines that serve as migrational cues (113–117). Therefore, altered chemokine receptor and adhesion molecule expression can affect the migrational properties of Treg and impact on their ability to access sites of chronic inflammation. Such attenuated Treg migration can in turn lead to sustained inflammation and increased risk of inflammation-driven cancer in the Treg inaccessible areas, owing to reduced frequency and suppressive activities.

The crucial role of chemokine-receptor-dependent migration in functional regulation is demonstrated in several experiments including a mouse model of colitis and IBD, where CCR4-deficient Treg had impaired migration to the mesenteric lymph nodes and therefore failed to prevent colitis (118). In other settings, a number of chemokine receptors including CCR2, CCR4, CCR5, CCR6, CCR7, and CXCR3 have been implicated in the selective and preferential recruitment of Treg to sites of chronic inflammation and/or tumors (115, 119, 120), thus indicating that alteration in chemokine receptor patterns or blockade of chemokine receptor signaling would have a significant impact on their migration and immuno-regulatory activities. Tumors and their associated stroma are known to express elevated levels of specific inflammatory chemokines, which serve to chemoattract various leukocytes including Treg (121, 122). Although the overall recruitment is also significantly influenced by the type of chemokine receptors expressed by the leukocytes, Treg, especially the “inflammation-seeking” phenotype usually up-regulate multiple chemokine receptors (116), which allow them access to a variety of tumors, where they preferentially accumulate (119). Some studies demonstrate that disruption of key chemokine receptor signaling axes such as CCR4 or CCR5, or the depletion of chemokine receptor-specific Treg can significantly inhibit their migration and prevent accumulation in tumors (123, 124), thus influencing the overall prognosis. Conceivably, while impaired migration and reduced Treg access to tumors would be an awesome advantage in the majority of cancer settings where they impinge on anti-tumor immune responses, it may however be a major setback in certain other settings, which require Treg to limit excessive immune responses, such as in IBD and chronic virus infections.

### Loss of Treg (Impaired Treg induction or Treg deletion)

In certain disease settings, physical deletion of Treg can result in reduced frequencies. For instance, it is postulated that by virtue of their activated nature, Treg express higher levels of CCR5 and CXCR4, the co-receptors for HIV-1 thus making them preferential targets for HIV-1 infection (125, 126). Since Foxp3+ Treg represent a high proportion of CD4+ T cells (up to 50%) found in mucosal lymphoid organs of HIV-infected individuals (60), it is plausible that HIV infection can subsequently lead to significant depletion of these cells and impaired immuno-regulatory functions (127–129). Furthermore, Treg also express both Fas and Fas ligand and can be targeted and killed by effector T cells without necessarily being infected. Several studies indicate that myeloid dendritic cells (mDCs) can contribute to Treg induction by promoting conversion of conventional CD4+ T cells into Treg (130, 131). However, it has been shown that *in vitro* HIV infection of mDCs not only impairs their capacity to induce Treg but can also trigger preferential targeting and killing of Treg via a caspase-dependent pathway (132), thus contributing to numerical loss of Treg. Changes in the levels of chemokines expressed within certain tissues, together with diminished levels of TGF-β and IL-2 can also result in the loss of Treg in that particular organ. For example, altered expression of ligands for CXCR3, CCR4, and CCR7 was associated with a loss of Treg in lymph nodes during simian immunodeficiency virus (SIV) infection (133). Other mechanisms for reduced Treg frequencies may include increased apoptosis, reduced proliferation and survival, as well as impaired peripheral Treg induction. As discussed earlier, Treg may also be lost by conversion to exFoxp3 T cells under certain inflammatory cytokine milieu.

## Imbalances in Immuno-Regulatory and Immuno-Stimulatory Processes Can Cause Cancer

**Figure 4:**
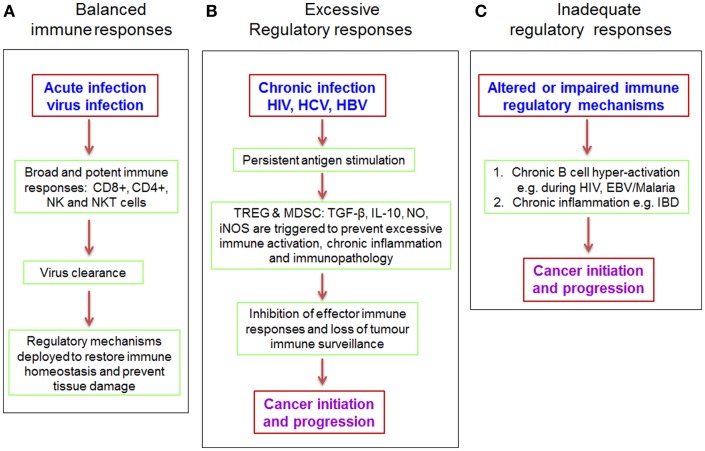
**Dysregulated immune responses create a microenvironment suitable for cancer initiation and progression**. Perturbations of the balance between effector and regulatory immune responses are often the cause of chronic inflammation and increased risk of cancer (146). Under normal circumstances **(A)**, the immune system mounts a potent and broadly directed immune response following acute virus infection. This immune response, comprising both the innate and adaptive components leads to effective virus clearance and stable memory cell formation, which is effective at rapidly countering subsequent infections. Under this scenario, regulatory mechanisms kick in to prevent tissue damage after virus clearance. However, in the case of persistent antigenic stimulation **(B)** such as caused by HIV, HCV, and HBV, there is continuous generation of effector immune cells that are incapable of clearing the pathogen. This leads to a state of chronic inflammation that in turn triggers regulatory pathways such as increased production of suppressive cytokines and recruitment of Treg and MDSC to dampen excessive immune responses and prevent tissue damage (136). But as fate would have it, such potent regulatory responses also inhibit anti-tumor effector responses leading to loss of tumor immune-surveillance and subsequently cancer initiation and progression. In certain contrasting scenarios such as IBD, impaired regulatory mechanisms **(C)** can result in chronic inflammation, which initiates carcinogenesis. Furthermore, uncontrolled B cell activation during HIV and EBV infections is associated with increased risk of non-Hodgkin’s lymphoma (147). NO, nitric oxide; iNOS, nitric oxide synthase.

Increased risk of cancer is often associated with poorly regulated immune responses (Figure 4) constituting unresolved inflammation as a result of perturbations in the balance of tumoricidal and tumorigenic activities (134, 135). Treg play a crucial role in maintaining optimum balance between these two arms of the immune response and persistent viruses are known to trigger production of IL-10 and TGF-β (136) to ensure induction and maintenance of adequate numbers of Treg in circulation. In some cases, viruses express homologs of immunosuppressive cytokines or cytokine receptors, such as the well-described human cytomegalovirus (HCMV)-IL-10 and EBV-IL-10 homologs (137, 138), which allow them to directly influence Treg induction or modulate the immune system via other mechanisms including impaired production of pro-inflammatory cytokines and chemokines, as well as MHC class II down-regulation (136). As mentioned earlier, viruses can also promote Treg induction by disrupting the normal activation cascade of dendritic cells and other antigen presenting cells. Furthermore, inflammatory micro-environments are enriched with type 2 macrophages (M2) and MDSC, which also enhance recruitment of Treg, besides directly suppressing antigen-specific effector T cells (19, 139, 140). Additionally, antigen-specific CD8+ Treg are frequently detected in chronic HIV (141, 142), HCV (57, 143), and herpes virus infections (144, 145). The increased numbers of Treg and other immunosuppressive mechanisms serve to actively prevent excessive immune activation and the associated immunopathology, but by so doing, they block antigen-specific effector immune responses that are essential for clearing the pathogen and for tumor immune-surveillance. The resulting immune impairment allows chronic pathogen persistence and an overwhelming state of recurrent inflammation, thus favoring cancer establishment.

Besides the direct disruption of tumor immune-surveillance, establishment of chronic inflammation creates a suppressive tumor-promoting microenvironment, which is enriched with IL-10, TGF-β, and other pro-inflammatory cytokines such as IL-17, known to be angiogenic and to contribute to tumor cell survival and growth (148, 149). In the presence of IL-6, TGF-β can further up-regulate ROR-γt expression leading to enhanced Th17 differentiation (150–152) and increased risk of cancer progression (148, 153–155). Moreover, as discussed earlier, Foxp3+ Treg in certain inflammatory environments can express IL-17, which together with hypoxic conditions could play a role in generation of cancer initiating cells (156). As highlighted in earlier sections, inflammatory environments can also induce phenotypic and functional impairments in immuno-regulatory cells thus leading to dysfunctional immune-regulation and increased risk of cancer. However, whether increased incidence of cancer in individuals with chronic virus infection and inflammation is due to increased suppression of tumor immunity as a result of increased frequency and suppressive activity of immuno-regulatory cells, due to failure of regulatory cells to prevent excessive immune activation and inflammation, or due to enhanced oncogenic potential of the carcinogen remains a subject of intense debate. In this review, I will focus on the contradictory roles of immuno-regulatory cells where they can cause cancer by either exerting potent suppression of effector immune responses that inhibit tumor immune-surveillance (e.g., during chronic virus infections) or through their functional impairment and inability to execute effective suppression of pathogenic effector immune responses (e.g., during IBD).

## The Delicate Balance between Immunity and Regulation in HIV Infection and Disease

Although HIV can exist in latent reservoirs for many years, it is a chronic persistent virus characterized by the continuous presence of infectious virus and thus chronic immune activation, persistent inflammation, and concurrent CD4+ T cell loss are all observed (157–159). Thus, increased numbers of fully functional regulatory mechanisms become necessary to counteract the ongoing inflammatory processes. However, Treg, which are instrumental in counteracting immune activation and inflammation by actively suppressing effector immune responses can also be detrimental by inhibiting T cell responses that control HIV replication. An appropriate immune response must therefore not disturb this delicate balance, by aiming to maximize the “good” immune responses, which control the virus while minimizing the “bad” immune responses that cause pathology.

Although still a subject of intense debate, a number of studies have demonstrated increased frequencies of Treg during HIV-1 infection and more especially in the chronic stages that mark progression to AIDS (128, 160–163). Studies looking at tissue distribution revealed accumulation of Treg at sites of HIV infection and replication such as the gastrointestinal mucosa and lymph nodes (59, 60, 128, 164). With such increased frequencies and especially given the suppressive role of Treg, it follows that progression of HIV-1 infection to chronic disease could in fact be a consequence of suppressed T cell function. Indeed, robust CD8+ and CD4+ T cell responses (characterized by high proliferation, IFN-γ production, and cytotoxicity), which correlate with HIV control in a minority of infected people usually diminish during chronic infection, coinciding with increased Treg numbers. Depleting Treg was shown to restore the *in vitro* effector immune functions of these cells (59, 165). Lower levels of Treg and a corresponding higher level of HIV-specific T cell responses have been observed in individuals who naturally control HIV-1 in the absence of HAART, i.e., the LTNPs and Elite controllers (160). Furthermore, depletion of Treg in cord blood samples of HIV-exposed uninfected neonates (166) was shown to augment both CD4+ and CD8+ HIV-specific T cell responses. These findings, together with the observation that Treg frequencies are reduced in HIV-infected patients on HAART (160, 164, 167, 168) provide compelling evidence that Treg impinge on immune control of HIV and strongly support immunotherapeutic interventions that reduce their numbers or impair their functions.

Whereas depleting Treg or interfering with their suppressive function might seem plausible in the context of immune function restoration, in fact several studies indicate that reduced Treg frequencies correlate with increased immune activation, which is in turn significantly associated with higher plasma viral loads (169, 170). Treg can therefore prevent collateral damage during chronic HIV infection by limiting immune activation, while at the same time reducing the pool of activated CD4+ T cell targets that would become susceptible to HIV infection. Accordingly, it is thought that the high frequencies of Treg found in highly exposed persistently sero-negative (HESN) individuals (171) and in the *in utero* HIV-exposed uninfected neonates (166) contribute to resistance to HIV infection by significantly reducing the numbers of activated target CD4+ T cells. These studies suggest that Treg may be beneficial at least to some extent, not only in HIV-infected individuals where they could limit immune activation, but also in highly exposed individuals with a greater risk of HIV infection. However, given that LTNPs and elite controllers exhibit both lower levels of immune activation and lower Treg frequencies (172), while at the same time mounting robust HIV-specific immune responses that inhibit virus replication, it is plausible to suggest that Treg are dispensable in HIV immunity, although caution must be exercised as LTNPs and elite controllers represent a very small proportion of HIV-infected individuals, in whom protective HLA alleles are over-represented.

Contrary to these findings, many studies document persistence of immune activation in the presence of elevated Treg frequencies, suggesting that perhaps the suppressive activities of the Treg found in chronic HIV infection are not sufficient to completely reverse the state of chronic immune activation. Indeed, it has been demonstrated that higher frequencies of Treg exist in HIV-infected individuals with progressive disease (173), but their ability to suppress HIV-specific T cells is significantly reduced, which in turn leads to inability to control HIV-associated aberrant immune activation (111, 161, 174). This is in fact discredited by studies demonstrating the existence of functionally suppressive Treg in progressive HIV-1 disease (59, 165, 175), hence suggesting that failure to reduce immune activation may be due to overwhelming levels of persistent stimulation rather than functional impairment of Treg. Thus, it is possible that high Treg frequencies found in chronic HIV infection are a result of failed attempts to reduce the state of chronic persistent antigenic stimulation (176, 177).

Faced with this paradox, timings of when to initiate interventions remain critical to achieving desirable outcomes. Whereas, immune-based therapies aimed at increasing the frequencies of Treg such as IL-2 therapy may only serve to suppress anti-HIV immunity and provide more targets for HIV, and thus not offer clinical benefit earlier in HIV infection (178, 179), they might indeed become useful during the chronic stages in order to limit immune activation (169, 170). Conversely, depleting Treg during the early stages of infection will allow for generation of robust immune responses capable of controlling virus replication and preventing establishment of latent reservoirs (126).

## HIV-Associated Immune Dysfunction Predisposes to Malignancies

The existence of a few HIV-infected individuals with robust HIV-specific immune responses who maintain very low virus loads for many years without treatment and only progress to AIDS following viral immune escape demonstrates constant immune-surveillance that keeps the virus in check. In these individuals, a normal balance between the effector and regulatory immune responses exists, whereby effective immune responses occur without excessive immune hyper-activation that causes T cell exhaustion and functional impairment. However, in a majority of HIV-infected people, the immune system does not control virus replication, leading to continuous immune stimulation with high antigen loads and generates a large pool of immune-effector cells that are by far inadequate in controlling the virus. This can be either due to anergy, functional exhaustion, or immune escape (157), as described earlier. Furthermore, HIV directly infects CD4+ T cells and this leads to the progressive diminution of T helper functions and immune incapacitation that marks progression to AIDS.

Besides these, a number of immuno-regulatory mechanisms triggered to prevent immune activation and inflammation also suppress immune-effector functions and sustain chronic virus persistence. For example, during chronic HIV infection, the expansion of Treg (180) with potent suppressive activity within mucosal tissues not only contributes to persistence of HIV, but also reduces immune vigilance and predisposes to HPV and cervical cancer. Moreover, HIV-1 gp120 has recently been shown to induce IL-6 and a concomitant expansion of MDSC (181), which contribute to immune suppression by modulating cytokine and cellular responses as well as inducing the differentiation and expansion of Treg (182). Large amounts of B cell activation-associated cytokines such as IL-6 and IL-10 are produced during chronic HIV infection and can also increase the numbers and suppressive capacity of MDSC leading to further suppression of effective immune responses. Indeed, higher levels of MDSC are associated with chronic progressive HIV disease (183). The decline of both IL-6 levels and Treg numbers following HAART-mediated immune restoration strongly supports their role in immune modulation during HIV-1 progression. A highly immunosuppressive environment with increased numbers of Treg, MDSC, and suppressive cytokines such as IL-10 and TGF-β is strongly associated with increased risk of cancer (19, 182, 184, 185). Thus, HIV-1 can be classified as indirect carcinogen that perturbs immune balance through immune suppression and a concomitant loss of tumor immune-surveillance to set the stage for oncogenic tumor viruses (186).

Another consequence of HIV-driven impairment of the immune system is the hyper-activation and uncontrolled proliferation of B cells, which not only favors secondary infection by oncogenic viruses (187) such as KSHV and EBV but also increases significantly the potential of chromosomal translocations and oncogenic mutations. A few studies have linked HIV infection with chronic B cell hyper-activation (147, 188) and lymphomagenesis, for example, increased incidence of Burkitt lymphoma in HIV-infected individuals or those persistently exposed to *Plasmodium falciparum* in malaria endemic regions where their B cells are in constant stimulation by these antigens (189). HIV-1 can also act directly via gp120 to induce B cell activation and subsequent development of lymphomas (190). Moreover, incorporation of CD40L into HIV virions stimulates B cell activation via interactions with CD40, resulting in production of B cell activating cytokines such as IL-6, IL-8, IL-10, and GM-CSF (191, 192). Indeed, HIV-associated lymphomas are often the aggressive B cell lymphomas, directly supporting a role for HIV in altering the B cell phenotypic and proliferative characteristics.

Therefore, the profound T cell dysfunction, progressive depletion of CD4+ T cells, B cell hyper-activation, together with the increased immuno-regulatory mechanisms all collude to actively impede tumor immune-surveillance and create a permissive environment for cancer initiation and progression. This is a classic example of a “vicious cycle of immune responses” where an effector immune response to a pathogen (in this case HIV) is induced during the initial stages of infection, but somehow fails to eliminate the pathogen, and regulatory mechanisms are triggered in order to restore immune balance and limit excessive inflammation and pathology, yet such regulatory mechanisms actively suppress the anti-tumor immune-surveillance processes and predispose to increased risk of cancer.

### HIV-associated malignancies

Cancer is a complex multistep process involving many molecular events, which together with the carcinogen or oncogenic virus infection work in concert to generate a transformed cellular phenotype. However, immune response is an important extrinsic factor that determines whether or not cancer occurs following exposure to potential carcinogens. While the immune system of healthy individuals limits proliferation of pre-malignant cells by recognizing and deleting cells that express potentially oncogenic viral proteins, these pre-transformed cells go unchecked and become malignant in immuno-compromised individuals, hence the increased incidence of cancer in transplant patients and those with congenital or secondary immunodeficiency disorders. HIV is not directly oncogenic but it is significantly associated with several lymphoid malignancies known to arise in immuno-compromised individuals who become infected with oncogenic viruses such as HPV, EBV, or KSHV (7). Surveillance data estimates the risk of developing NHL at 60- to 200-fold in people with progressive HIV disease compared to the uninfected population, while that of Hodgkin lymphoma (HL) is 8- to 10-fold, thus supporting the active role of the immune system in controlling cancer. Plausibly, HIV-mediated immune dysregulation contributes to immune escape of these viruses thus allowing for proliferation and emergence of stable populations of virally transformed cells that are not efficiently recognized and eliminated by the host’s immune system (187, 193, 194). A wide body of literature documents several AIDS-defining malignancies in the pre-HAART era, but for the purposes of illustrating how immune dysregulation sets a microenvironment conducive for cancer development, this section will draw examples from HIV-associated predisposition to cervical cancer and KS.

### HIV-1, KSHV, and Kaposi sarcoma

The non-redundant role of host immunity in the control of viral cancers is well-illustrated by KS, which is more prevalent in untreated HIV/AIDS patients, mainly due to immunosuppression (195). KSHV was discovered as the causative agent of KS in 1994 (196), however, infection with this virus alone is not sufficient to cause KS in healthy immuno-competent individuals. Indeed, the incidence of KS in the general population remains very low (around 1/100,000), but increases dramatically to around 1/20 amongst HIV-infected people (197) and almost 1/3 HIV-infected homosexual men in the pre-HAART era (198). Furthermore, countries in which KS was endemic before the AIDS epidemic have seen a sharp increase in the incidence, with almost half of HIV-infected individuals who acquire KSHV infection going on to develop KS (199). However, within the endemic areas or in the high risk groups, most HIV-negative KSHV-infected individuals do not develop KS, indicating that HIV-associated immune impairment predisposes to KS development.

### HIV-1, HPV, and cervical cancer

Human papilloma viruses are the main etiological factor for cervical cancer (200). Of these, HPV-16 and HPV-18 are linked with cervical and anogenital cancers hence are classified as high risk genotypes. As with other cancers, the immune system is central in the pathogenesis of HPV and cervical cancer. In immuno-competent individuals, robust HPV-specific immune responses comprising B and T cells are generated and these correlate with spontaneous resolution of HPV (201, 202), demonstrating that host immunity can be sufficient to clear HPV infection. In particular, a Th1 cytokine profile is instrumental in HPV clearance and prevention of viral persistence. Thus, detection of both humoral and cellular responses including T helper cells induces regression of cervical lesions (203, 204), whereas T helper cell impairment leads to cancer development (205). Natural killer cells also play a protective role by directly lysing HPV-infected cells and initiating regression of squamous intraepithelial lesions (SIL) (206, 207).

Despite the existence of strong HPV-specific immune responses in HPV-infected individuals, progression to HPV-associated malignancies does occur in some individuals due to escape from immune-surveillance caused by immune dysfunction as discussed earlier. Central to this is the systemic enrichment of Treg, which correlates with HPV persistence and is frequently detected in patients who develop high grade cervical intraepithelial neoplasia (208, 209). Furthermore, mucosal enrichment of Treg, which is often associated with diminished cellular immunity in the cervical mucosa has been observed and is linked with the severe forms of cervical carcinoma (210, 211). Higher frequencies of HPV-specific Treg are found in the stroma, intraepithelial tissues and tumor draining lymph nodes of cervical cancer patients where they suppress alloreactive CD4+ responder T cells (212, 213). Depletion of Treg in the *in vitro* experiments resulted in increased production of IFN-γ. Besides enhanced Treg-mediated immunosuppression, the profound immune dysfunction resulting from HIV-1 infection and the concomitant loss of CD4+ T cells collude to create an environment permissive for HPV persistence and cervical cancer. This can be directly deduced from the increased incidence of cervical cancer and prolonged persistence of SIL in immunosuppressed women with progressive HIV disease (214–216). In fact, cervical cancer was designated as an AIDS-defining illness in 1993 (217), strongly implicating HIV-driven immune impairment as a major factor favoring the progression from HPV infection to cancer development.

### Immune restoration or HIV suppression reduces HIV-associated malignancies

There is consensus that HIV-associated malignancies arise mainly due to loss of immune-surveillance caused by a dysfunctional immune system. Indeed, the severity of these malignancies correlates positively with the degree of immune impairment as measured by the extent of CD4+ T cell depletion and HIV viral burden. Moreover, the incidence of AIDS-defining malignancies has significantly reduced since the wide-scale implementation of HAART, strongly suggesting better immune control following reconstitution by HAART or perhaps a direct impact of HAART on the replication of EBV, HPV, and KSHV. Therefore, it seems that interventions which limit virus production and prevent chronic antigenic stimulation can effectively reduce immune activation and inflammation, restore effector immune functions through homeostatic equilibration of immuno-stimulatory and regulatory mechanisms, and lead to reduced incidences of HIV-associated malignancies. Recent studies indicate that the increased frequency and suppressive function of Treg observed during chronic HIV infection decreases significantly following HAART initiation (167). This is accompanied by reduced levels of immune activation and enhanced immune-effector functions, which are in turn associated with decreased prevalence and increased regression of cervical lesions in HAART-treated HPV-infected patients (218–220), thus supporting a role for immune reconstitution in the control of HPV and associated cancers. These observations provide evidence for a strong causative link between HIV-mediated immune dysregulation and the onset of HIV-associated cancers (NHL, KS, and cervical cancer) whose incidence has reduced significantly since the introduction of HAART.

## HCV/HBV-Driven Immune Dysregulation Predisposes to Hepatocellular Carcinoma

Unlike HIV, which directly targets the immune cells (CD4+ T cells) causing their deletion and loss of T helper functions, HBV and HCV target the liver and replicate in hepatocytes. These viruses have also evolved multiple mechanisms to escape immune elimination and can establish chronic persistence and replicate in infected hosts for many years. Epidemiological studies indicate a strong link between chronic HBV/HCV persistence with the development of liver disease, initially manifesting as chronic hepatitis, and leading on to nodular fibrosis that can progress to cirrhosis and eventually hepatocellular carcinoma (HCC). These processes are characterized by inflammation and oxidative stress owing to the influx of several cell types including NK, NKT, and PMN leukocytes, which accumulate in inflammatory lesions in the liver and contribute to inflammation and liver damage. In a majority of infected individuals, robust and poly functional T cell responses are generated causing clearance of acute infection, while in a minority of those infected, both low frequencies and narrowly focused virus-specific CD8+ T cell responses in the liver correlate with persistent chronic infection and increased risk of HCC (221). Furthermore, defects in HBV-specific CD8+ T cells characterized by exhaustion and increased expression of pro-apoptotic mediators have been reported (222). Thus, although virus-specific lymphocytes can be readily detected in inflammatory lesions in the liver, they are often defective and not sufficient to clear virus infection (223). Moreover, weaker CD4+ T cell proliferative responses have been reported (224).

Infection with HBV and HCV is known to induce IL-10 and TGF-β (72, 73), which in turn induce the expansion of Treg to maintain a tolerogenic environment in the liver. HCV-specific impairment of dendritic cell function can also lead to increased numbers of Treg, and these have been found in both the blood and liver of patients with chronic HBV and HCV infection and HCC (185), where they correlate with *in vitro* suppression of antigen-specific effector responses (225). These effector responses were enhanced by depleting Treg (54, 226). Overall, immune function restoration and inhibition of viral replication following treatment with anti-HBV drugs is associated with diminished Treg expression (227). Thus, persistence of weak, defective, and narrowly directed T cell responses coupled with high numbers of immune-regulatory cells and increased levels of suppressive cytokines act to promote chronic liver disease and progression to HCC. Indeed, patients with HCC often have increased Treg numbers in blood and within tumors, and the tumor-infiltrating CD8+ and CD4+ T cells have been found to be dysfunctional (228), suggesting a possible link between immune disruption and the pathogenesis of HCC. Other factors such as chronic unresolved inflammation can further support tumor growth via induction of angiogenic and tumor survival signals (229).

## Immune Dysregulation in Inflammatory Bowel Disease and Colorectal Cancer

Inflammatory bowel disease is characterized by an uncontrolled, microbe-induced chronic inflammatory state that increases the risk of colorectal cancer (CRC) by twofold (8, 9). These chronic inflammatory responses also drive carcinogenesis of colitis-associated cancer (230). Various cell types infiltrate the inflamed mucosa including MDSC, M2 macrophages, and Th17 cells, which promote tumor growth, and NK and CD8+ T cells, which either target and destroy or inhibit proliferation of CRC cells. These effects are mediated by cytokines such as IL-17A, IL-21, IL-6, and TNF-α that create a tumor-permissive environment versus IFN-γ, which exerts tumor-suppressive functions (231). IFN-γ protects from carcinogenesis by activating cytotoxic T cells as well as increasing the susceptibility of pre-malignant cells to cell-mediated cytotoxicity, thus IFN-γ-producing Th1 cells correlate with increased immune-surveillance and better prognosis in CRC patients (232).

Although IL-4- and IL-13-producing Th2 cells have been associated with increased tumor growth in humans (233) and in animal models using IFN-γ−/−and IL-4−/− deficient mice (234, 235), Th17 cells seem to be the most aggressive orchestrators of chronic inflammation during IBD and have a significant role in the initiation of CRC. This has been linked to IL-23, a cytokine known to induce high numbers of Th17 cells and a concomitant accumulation of pathogenic IL-17A+ IFN-γ+ effector T cells, which cause intestinal pathology and correlate with poor prognosis in CRC (153, 236–238). Indeed, high frequencies of activated Th17 cells together with their signature cytokines are found in the intestinal and serum samples of patients with IBD, and also within the colon and blood samples from patients with CD. Furthermore, IL-23-mediated accumulation of IL-17+IL-22+ innate lymphoid cells (ILCs) in inflamed colons is associated with development of invasive colon cancer (239–241), while increased frequencies of IL-17+ILCs are often found in the intestines of patients with CD (242). The tumor-promoting feature of Th17 cells largely arises from secretion of large amounts of IL-17, which in turn induces expression of pro-inflammatory factors such as TNF-α, IL-6, IL-1, and iNOS, known to play a role in CRC pathogenesis (243). Mice that are deficient in ROR-γt, the transcription factor of Th17 cells were shown to be resistant to chronic inflammation in models of colitis (244). Thus, immuno-regulatory pathways capable of limiting the induction and function of pathogenic Th17 effectors cells are required.

### Treg play a critical role in the pathogenesis of IBD and CRC

The pro-tumoral role of Treg in cancer establishment and progression is well-documented, and in fact a number of interventions that deplete Treg lead to improved prognosis of cancer patients. Furthermore, Treg depletion increases vaccine-mediated anti-tumor immunity (245) and can lead to eradication of established experimental tumors (210, 246). However, Treg play such a critical role in the maintenance of normal gut mucosal immunity by preventing chronic inflammatory responses to food antigens and commensal microflora (247), that inhibition of their function is associated with development of IBD (11, 12). Most astoundingly, increased infiltration of Treg in CRC correlates with a favorable prognosis (10), with several studies in experimental animal models providing evidence that Treg can prevent establishment of CRC (248, 249). This is thought to be through initiation of potent immuno-regulatory functions that prevent chronic inflammation, which would otherwise predispose to cancer establishment and growth. Under normal homeostatic conditions, high frequencies of Treg are found in the gut as it is a preferential site for peripheral Treg induction due to the abundant commensal micro-biota and CD103-producing DCs, which are specialized in inducing the differentiation of Treg from naïve CD4+ T cells (250, 251). However, inadequate regulatory functions are a major characteristic defect during IBD, suggesting alterations in the induction, maintenance, or even suppressive function of Treg. This section highlights some of the mechanisms of immune dysregulation that exacerbate the inflammatory state of IBD to set a stage for CRC.

### Treg induction and function are impaired in IBD and CRC

Impaired frequency and function of Treg is one of the mechanisms of immune dysregulation that plays a central role in the pathogenesis of IBD. This is strongly associated with IL-23, a cytokine whose expression is increased in several human cancers including CRC (252). IL-23R signaling suppresses both the differentiation of Treg and IL-10 production by T cells, hence leading to intestinal pathology (236). Such pathology could be prevented by transfer of Treg or administration of Treg-related cytokines such as IL-10 and TGF-β1 (253). TGF-β signaling in tumor-infiltrating lymphocytes is associated with reduced tumor growth in animal models of CRC (254). Crucially, the frequency of Foxp3+ Treg in the colon increases in the absence of IL-23R signaling, indicating a role for IL-23 in controlling the induction and expansion of Treg (255). Since Treg are a source of both IL-10 and TGF-β, the key cytokines in immuno-regulation, it is plausible that IL-23-driven loss of Treg contributes significantly to immune dysregulation by overriding the immunosuppressive pathways in the intestine and favoring IBD and CRC development via generation of pathogenic Th17 effectors cells. Besides reduced numbers, Treg in IBD show altered phenotype and function, attributed to the local cytokine milieu arising from chronic inflammation of the intestinal mucosa. Perhaps, normal Treg in circulation migrate to the lamina propria during active inflammation in order to maintain homeostasis, but on encountering various cytokines within the inflamed mucosa, they undergo phenotypic and functional modifications turning into dual inflammatory and regulatory Foxp3+IL-17+ Treg, which produce large amounts of IFN-γ and IL-17, and moderate amounts of TNF-α and IL-2 (102, 103).

### IL-10 protects against IBD and CRC

IL-10 deficiency increases susceptibility to IBD-associated CRC, where it is associated with poor prognosis (256). Mice lacking IL-10 were shown to be highly susceptible to colitis-associated CRC following *Helicobacter hepaticus* infection, and this could be prevented by exogenous administration of IL-10 (257–259), further demonstrating a critical role for IL-10 in the pathogenesis of CRC. It is thought that IL-10 deficiency leads to elevated levels of TNF-α, IL-6, and IL-17, which in turn allow persistence of chronic inflammation (260) thus promoting tumor growth.

### Bi-functional immune-effector cells can promote IBD and CRC

Intriguingly, a single cell type can exhibit bi-functional immune characteristics by co-producing effector and suppressor cytokines, thus may have the potential to exert both tumor-promoting and tumor-suppressive functions, depending on the microenvironment. For example, as mentioned above, CD8+ T cells express cytotoxic molecules, which kill CRC cells in addition to secreting IFN-γ, which augments the anti-tumor response (261, 262). However, in some cases of IBD, infiltration of CD8+ T cells does not correlate with improved prognosis (263) and this is linked to elevated perforin and granzyme levels, which sustain the tumor-promoting chronic inflammation (264). Accordingly, perforin deficient mice develop less severe colitis and much fewer tumors in experimental models of colitis-associated CRC (265). Similar bi-functional characteristics have been observed in NKT cells, which exert protective cytotoxic functions, but also secrete Th1, Th2, and Th17 cytokines that could act as enhancers or suppressors of tumor immunity. Increased infiltration of IFN-γ-secreting NKT cells correlates with tumor immunity, which is reflected in increased disease-free survival of CRC patients (266, 267). Conversely, Th2 NKT cells that secrete the immunosuppressive cytokine IL-13, may contribute to colitis-associated CRC (268, 269). These studies demonstrate that CD8+ T cells and NKT cells can simultaneously exert pro-tumoral and anti-tumoral responses, and that perhaps pro-tumoral responses predominate during progressive IBD and CRC. Arguably, intervention strategies targeted at these bi-functional effector cells may result in undesirable outcomes.

## Interventions

As discussed earlier, some settings such as inflammatory autoimmune diseases will require interventions that boost the immuno-regulatory arm of the immune response. Such may include immunotherapeutic agents that expand Treg numbers and enhance suppressive function to effectively curtail chronic inflammation. Therapeutic vaccines to restore immune tolerance could benefit from adjuvants that induce adaptive Treg without generating functional effector cells (270). Other measures such as restoration of TGF-β and IL-10, together with IL-2 administration can help to maintain Treg numbers and Foxp3 expression, thus sustaining functional regulation. In other inflammatory settings such as IBD and colon cancer, measures that enhance Treg differentiation and expansion and restore suppressive function, for example, blockade of IL-23 signaling with the concurrent depletion of IFN-γ and IL-2 to impede generation of pathogenic exFoxp3 Treg might be desirable. Additionally, induction of stable expression of site-specific homing and chemokine receptors in Treg can confer the ability to migrate to preferential sites of chronic inflammation, for example, CCR4 for migration to the lung airways during allergic inflammation, CXCR4 for migration to the bone marrow, and CCR4/CCR9/CD62L/α_4_β_7_/α_E_(CD103)β_7_ for migration to the intestinal mucosa of IBD patients. However, in cases where immuno-regulatory responses are detrimental then immune deregulation interventions are required. Such can include administration of cytokines and/or antibodies that inhibit Treg induction and expansion, suppressive function, and recruitment via blockade of chemokine receptors (124). Interventions such as concurrent CTLA-4 blockade and vaccination (271–273), combined CTLA-4 and PD-1/PD-L1 blockade (274), and Treg depletion (275–277) have been successfully used to ameliorate Treg-mediated immune pathologies and cancer. Measures to reverse exhaustion and restore immune function in chronic infections include blockade of the PD-1:PD-L1/PD-L2 pathway and MDSC development. PD-1/PD-L1 blockade restores HIV-specific T cell function *in vitro* (33, 34, 278), and clinical benefit is also documented in cancer patients (279, 280). In some instances, combined blockade of PD-1 and LAG-3 or PD-1 and TIM-3 synergistically improves T cell responses leading to better virus control (43, 45). Very recently, a study utilizing a mouse model of retrovirus infection showed that combining the blockade of inhibitory receptors PD-1 and Tim-3, together with Treg ablation was more efficient in reducing chronic virus load compared with either strategy on its own (281). Functional blockade, developmental inhibition, or physical deletion of MDSC was shown to enhance the efficacy of cancer vaccines in animal models (282–284).

## Conclusion

The role of the immune system in inflammation and carcinogenesis is highly influenced by the microenvironment, thus some disease settings can display unique characteristics where immuno-regulatory processes are highly beneficial to the host but in other cases quite detrimental and predispose to pathogen persistence and increased risk of cancer. This calls for tailor-matched interventions, which are quite promising, however caution must be exercised since blocking an inhibitory pathway might re-invigorate the immune system to achieve disease control on one hand, but exacerbate immune activation and inflammation on the other. Overall, the timings of these interventions will be crucial in order to achieve favorable outcomes.

## Conflict of Interest Statement

The author declares that the research was conducted in the absence of any commercial or financial relationships that could be construed as a potential conflict of interest.
